# DOCUMENTING REPORTS OF SUSPECTED ABUSE AND NEGLECT: THE EVALUATION OF A STANDARDIZED PATIENT NOTE

**DOI:** 10.1093/geroni/igae098.3951

**Published:** 2024-12-31

**Authors:** Amy Webster, Sara Webb, Nina Sperber, Kasey Decosimo, Jaime Halaszynski, Jennifer Koget, Jennifer Silva, Lena Makaroun

**Affiliations:** Durham VA Health Care, Durham, North Carolina, United States; Durham VA Health Care, Durham, North Carolina, United States; Durham VA, Durham, North Carolina, United States; Durham VA Health Care, Durham, North Carolina, United States; Butler VA Health Care System, Butler, Pennsylvania, United States; Veterans Health Administration, Pittsburgh, Pennsylvania, United States; Department of Veterans Affairs, Washington, District of Columbia, United States; VA Pittsburgh Healthcare System, Pittsburgh, Pennsylvania, United States

## Abstract

Elder abuse (EA) impacts 10% of older adults in the U.S. annually with devastating health consequences, yet few healthcare systems systematically document reports of EA in the electronic health record (EHR). We present acceptability and usability findings from the first of a two-phase Veterans Health Administration (VHA) quality improvement pilot of a standardized note template developed by the National Social Work Program for documenting reports of suspected abuse/neglect in the EHR. In spring 2024, we conducted a usability evaluation through semi-structured interviews with 22 staff from 3 sites including the following roles: 19 social workers, 1 nurse, 1 physical therapist, and 1 mental health case manager. Overall, participants found the note easy to access and use describing it as “really straightforward”, “simple”, and “self-explanatory.” Participants reported the standardized note made it easier to identify that a report was made and facilitated follow-up care for patients who needed it. Half of participants completed the note in ≤5 minutes. Staff in one location said that the template streamlined the reporting process, making it “quicker and easier.” Several staff suggested improving procedures to ensure patient privacy and safety when documenting. Modifications based on findings were incorporated to improve usability. Following the second phase of the pilot with 5 additional sites (ongoing), the refined note template will be disseminated for national use in VHA, creating opportunities for real-time monitoring of EA at a population level. Lessons learned from this initiative could inform other healthcare systems interested in systematizing clinical documentation for abuse/neglect reports.

